# Improved high temperature radiation damage tolerance in a three-phase ceramic with heterointerfaces

**DOI:** 10.1038/s41598-018-31721-x

**Published:** 2018-09-18

**Authors:** Kenta K. Ohtaki, Maulik K. Patel, Miguel L. Crespillo, Keyur K. Karandikar, Yanwen Zhang, Olivia A. Graeve, Martha L. Mecartney

**Affiliations:** 1 Department of Chemical Engineering & Materials Science, University of California, Irvine, Irvine, CA 92697-2575 USA; 2 Department of Mechanical, Materials & Aerospace Engineering, University of Liverpool, Liverpool, L69 3BX UK; 3 Department of Materials Science and Engineering, University of Tennessee, Knoxville, Knoxville, 37996-2100 TN USA; 4 Ion Beam Materials Laboratory, University of Tennessee, Knoxville, Knoxville, 37996 TN USA; 5 Department of Mechanical and Aerospace Engineering, University of California, San Diego, San Diego, CA 92093-0411 USA

## Abstract

Radiation damage tolerance for a variety of ceramics at high temperatures depends on the material’s resistance to nucleation and growth of extended defects. Such processes are prevalent in ceramics employed for space, nuclear fission/fusion and nuclear waste environments. This report shows that random heterointerfaces in materials with sub-micron grains can act as highly efficient sinks for point defects compared to grain boundaries in single-phase materials. The concentration of dislocation loops in a radiation damage-prone phase (Al_2_O_3_) is significantly reduced when Al_2_O_3_ is a component of a composite system as opposed to a single-phase system. These results present a novel method for designing exceptionally radiation damage tolerant ceramics at high temperatures with a stable grain size, without requiring extensive interfacial engineering or production of nanocrystalline materials.

## Introduction

Ceramics used in nuclear energy related applications experience extreme conditions of radiation and high temperatures. For example, in the case of nuclear fuel, such conditions lead to macroscopic swelling of the fuel and degradation of thermal conductivity. This puts a limit on higher burnups, thus decreasing the efficiency of the fuels. On a basic level, interaction of radiation with materials creates a Frenkel pair i.e. an interstitial and a vacant lattice site. At low temperatures these defects are not mobile and thus cause destruction of the crystalline order in ceramics, leading to amorphization. Thus, at low temperatures, radiation tolerance is attributed to a materials resistance to amorphization. Most of the radiation damage studies in the past have focused on studying ceramics irradiated at cryogenic temperatures to understand the fundamental defects created by radiation^1^. However real applications demand the understanding of material behavior under synergistic effects of radiation and high temperatures. Frenkel defects are mobile at high temperatures, leading to the formation of extended defects such as interstitial or vacancy dislocation loops and voids. Thus, at high temperatures the radiation tolerance is attributed to a materials’ resistance for nucleation and growth of these extended defects. On a macroscopic scale, these defects cause degradation of mechanical properties and thus limit the service life of materials.

The basic knowledge of materials science and engineering teaches us that in order to reduce the agglomeration of interstitials and vacancies at high temperatures, one must increase the sites (sinks) at which these defects can annihilate, thus rendering their contribution to radiation induced swelling negligible. Grain boundaries are the most common sinks that act as sites for annihilation of defects. Thus, research in the past has focused on, (1) increasing the grain boundary concentration by fabricating materials with an average grain size of 10–100 nm^2–5^, (2) carefully engineering interfaces in materials which will act as most efficient sinks^6^ and (3) fabricating thin film materials with nanoscale layers^7–10^. Although these approaches have led to a fundamental understand of defects interaction at interfaces, designing materials with these functional attributes for technological applications has seen limited success.

A fine grain size is expected to increase the radiation damage tolerance in polycrystalline materials since point defects generated during irradiation have only a short diffusion distance to reach grain boundaries that serve as sinks. Improved radiation damage tolerance has been demonstrated in a variety of nanocrystalline ceramics^2–4,7,11^. However, diffusion of interstitials to grain boundaries requires the grain sizes to be below 20 nm in single-phase ceramics at low temperatures. At high temperatures these single-phase nano-grained materials will undergo grain growth leading to a larger distance between grain boundaries, thus decreasing the concentration of available sinks. Fabricating multi-layer thin films requires specialized fabrication techniques which are difficult and expensive to scale up for the production of bulk materials.

Multiphase compositions can be used to limit grain growth by having few grain boundaries between similar phases in the microstructure, requiring coupled long distance diffusion from another grain of the same composition for that grain to grow^12^. Thus, multiphase materials should have the advantage of retaining a fine grain size even under irradiation at high temperatures. These composites can be fabricated with common bulk ceramic fabrication methods and do not require special techniques for large scale production.

This current research was sparked by the idea of using additional phases to increase the thermal conductivity of nuclear fuel, following the concept developed for inert matrix nuclear fuel using UO_2_ as a fissile component and a ceramic matrix phase to promote structural integrity^13^. Such fuels would then enable higher burnup with minimal microstructure evolution. Ceramic composites could also be used to transmute nuclear waste in fast reactors or as multiphase waste matrices to immobilize radioactive species in nuclear waste for final disposal^13^.

What is unknown, however, is whether multiphase polycrystalline ceramics exhibit differences in radiation damage tolerance compared to polycrystalline single-phase ceramics with a similar grain size. Do we always need to engineer interfaces with specific chemistries to obtain materials with greater radiation tolerance? Can we make an inherently radiation-susceptible material radiation-tolerant by incorporating it in a composite system? Improved radiation stability has been shown for metallic nano-composites compared to single-phase metals^14,15^.

In order to answer these questions, a model ceramic composite system comprised of equal portions of Al_2_O_3_, YSZ and MgAl_2_O_4_ phases was chosen. In this system, radiation damage behavior of the Al_2_O_3_ phase in single phase Al_2_O_3_ and in a three-phase composite was investigated since Al_2_O_3_ is well known for its susceptibility to void swelling as opposed to the MgAl_2_O_4_ and YSZ phases which are resistant to void swelling^16,17^.

## Results and Discussion

**Figure 1:**
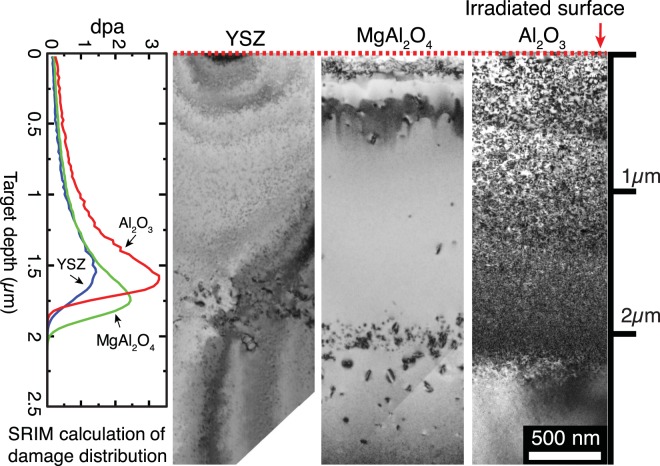
Cross section TEM bright field images of irradiated single crystals of YSZ, MgAl_2_O_4_ and Al_2_O_3_ from top surface to irradiated interior with Stopping and Range of Ions in Matter (SRIM) calculation^29^. SRIM calculations predict that the damage peak lies at ~1.6 μm from the irradiated surface and the damage depth is ~2 μm. Even though MgAl_2_O_4_ is known to be sensitive to ionizing and displacive irradiation spectra, only a small amount of dislocation loops was observed in this study. This must be due to ionizing radiation effect with high electronic to nuclear stopping power ratio whose influence becomes more significant for light elements^18^.

Figure 1 shows single crystals of each phase after 4 MeV Si^2+^ ion irradiation at 650 °C (fluence 10^16^ ions/cm^2^, flux 1 × 10^12^/cm^2^·s), which simulates the damage expected at high temperatures in a nuclear reactor from primary knock on atoms. These results in Fig. 1 demonstrate that the irradiation condition used generates a high enough concentration of point defects with sufficient mobility to form dislocation loops in the damaged region. It can be clearly seen how susceptible Al_2_O_3_ is to radiation damage compared to other two phases; i.e., dislocation loops form throughout the entire irradiated region for Al_2_O_3_. Due to this high damage susceptibility, Al_2_O_3_ was used in this study to compare the effect of a composite system to a single-phase polycrystalline system with respect to radiation damage at a high temperature.

**Figure 2:**
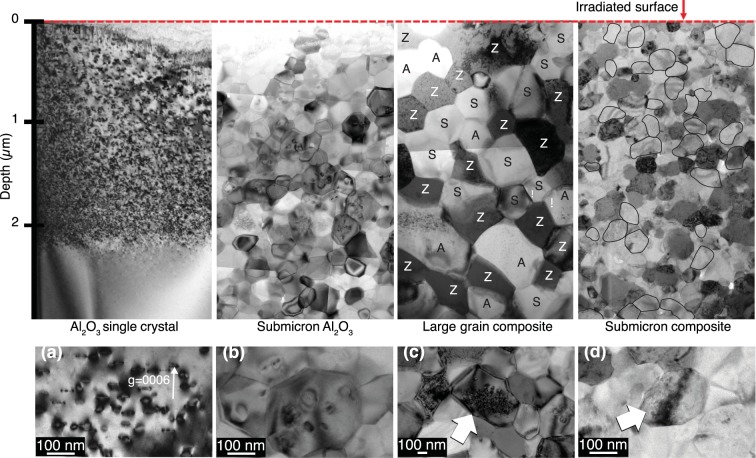
A comparison of TEM bright field cross section images of Al_2_O_3_ single crystal, submicron Al_2_O_3_, large grain composite and submicron composite after irradiation (A: Al_2_O_3_, Z: YSZ, S: MgAl_2_O_4_). The circled grains in the submicron composite are Al_2_O_3_. (**a**) Dark field image of single crystal Al_2_O_3_ near [10 $\overline{1}$ 0] with a g-vector [0006] at 1 μm from the surface. Dislocations lie on {0001} and {0 $\overline{1}$ 10} as reported^1,16^. (**b**) Dislocation loops in submicron Al_2_O_3_ clearly show dislocations loops even in small grains. (**c**) Arrow indicates a large Al_2_O_3_ grain with dense dislocation loops in large grain composite at the damage peak. (**d**) Arrow indicates an Al_2_O_3_ grain in submicron composite at the damage peak with salt and pepper contrast, but no dislocation loops.

Polycrystalline materials of single-phase Al_2_O_3_ with 290 ± 100 nm grains (submicron Al_2_O_3_), a three-phase composite with 260 ± 85 nm grains (submicron composite) and three-phase composite with 690 ± 200 nm grains (large grain composite) were prepared by spark plasma sintering (SPS). All samples were irradiated under the same conditions as the single crystals. Microstructures of the irradiated polycrystalline grains (Fig. 2) show no grain growth but the formation of dislocation loops in submicron Al_2_O_3_ throughout the damaged region. This damage was significantly lower than in the single crystal Al_2_O_3_, confirming that grain boundaries serve as sinks for point defects that form the dislocation loops. In the large grain composite, dislocation loops were only observed at the damage peak (~2 μm). In contrast, Al_2_O_3_ grains in the submicron composite contain no discernable dislocation loops, although in some grains a “salt and pepper” contrast may be due to point defect clusters. The lack of dislocation loop formation in Al_2_O_3_ for the submicron composite was verified for multiple samples and multiple tilts at different Bragg conditions.

**Figure 3:**
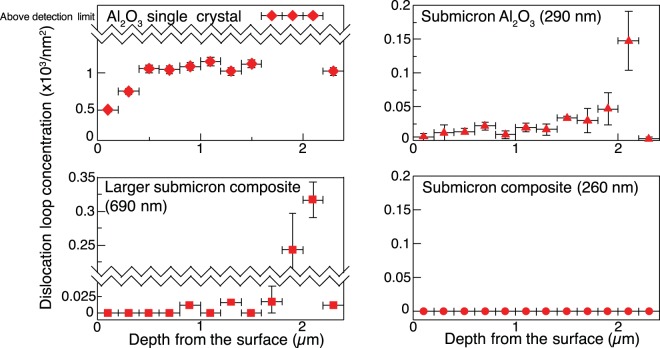
Areal dislocation loop concentrations with respect to the sample depth in Al_2_O_3_ single crystal, submicron Al_2_O_3_, Al_2_O_3_ phase in large grain composite and Al_2_O_3_ phase in the submicron composite. At the Al_2_O_3_ single crystal damage peak, dislocations are too concentrated to count. The error bars are the standard deviation generated from three different areas for the concentrations and the depth range used for each calculation.

The calculation of areal dislocation loop concentration with respect to irradiation depth based on TEM analyses using a series of random tilts is shown in Fig. 3. Efficient elimination of point defects during irradiation of nanocrystalline oxides has been shown to be effective for grain sizes well below 100 nm for a range of experimental conditions at relatively low temperatures^2–4^. In addition, near the grain boundaries of irradiated ceramics at high temperatures denuded zones have been observed that range from 20 nm to 1 μm, depending on the irradiation condition and the material^18–20^. However, this current work shows that by simply fabricating a composite system with heterointerfaces, the larger grain size in the large grain composite shows a similar reduction of dislocation loops as in the smaller grain submicron Al_2_O_3_ in the region before the damage peak. At the damage peak in the large grain composite, the grain size is not small enough to efficiently annihilate point defects. In contrast, for the submicron composite in Fig. 3, the introduction of a high concentration of heterointerfaces (as opposed to similar concentration of grain boundaries) generated essentially zero dislocation loops throughout the entire damage region.

Two hypotheses can explain the dramatically improved radiation damage tolerance for Al_2_O_3_ when present in a three-phase composite. (1) There is more effective annihilation at heterointerfaces than for single phase grain boundaries due to the increased structural and chemical disorder between the different phases, resulting in higher entropy grain boundaries. (2) Residual stress due to differences in thermal expansion between different phases can enhance diffusion to the heterointerfaces over grain boundaries. (Micro cracking was not observed.) Supporting the first hypothesis, simulations on thin amorphous intergranular layers in a polycrystalline metals suggest that interfaces with crystal disorder work better as sinks for point defects^21^. The high disorder of the heterointerfaces may accommodate a higher concentration of point defects. Prior research in nano-composite metals has shown that the grain boundary structure and, in particular, heterointerfaces serve as more efficient sinks for point defects during irradiation^8,22^. Moreover, differences in chemical potential can drive the migration of point defects to heterointerfaces^6^. For the latter hypothesis, we can look at estimations of the maximum residual stress. Al_2_O_3_ has anisotropic thermal expansion along its a-axis and c-axis due to its rhombohedral crystal structure generating maximum thermal stress at a grain boundary where the a-axis of one grain and the c-axis of the other are coincident. In the composite system, the heterointerfaces between Al_2_O_3_/YSZ and Al_2_O_3_/MgAl_2_O_4_ generate thermal stress three times higher than the maximum thermal stress in submicron Al_2_O_3_ (see Supplementary Table S2). The higher residual stress in the composite systems can enhance diffusion to the interfaces^23,24^ and increase the annihilation rate of point defects in the composite. Additionally, point defect annihilation has been demonstrated to be enhanced in high energy/non-equilibrium grain boundaries in single-phase nanocrystalline iron, explained by the excess free volume and disorder at the grain boundaries with strain near the grain boundaries^25^. The same phenomena are expected to be present at heterointerfaces in our three-phase system, but at a much larger and more stable grain size.

**Figure 4:**
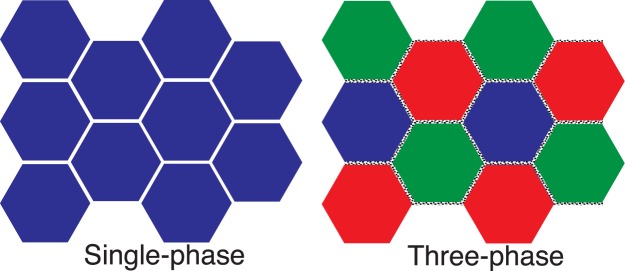
Schematic microstructures of a single-phase polycrystalline ceramic and a three-phase ceramic. Heterointerfaces with more disordered interfaces improve radiation tolerance for susceptible phases better than grain boundaries in a single phase polycrystalline.

While the contribution of each hypothesis to enhanced point defect annihilation at grain boundaries cannot be quantitatively distinguished, the effectiveness of using composite polycrystalline structures to dramatically enhance radiation damage tolerance has been established by this research. Improved radiation damage tolerance due to heterointerfaces is expected not only in a three-phase system but also for a two-phase system. However, three phases are the most effective for limiting grain growth (thus having a high concentration of grain boundaries). In addition, three phases will generate more heterointerfaces than a two-phase system. In summary, single phase nanocrystalline materials are known to be radiation damage tolerant, but their instability to grain growth during irradiation (even at cryogenic temperatures^26^) and at high temperatures precludes their use. Carefully engineered nano-crystalline/nano-layered systems and grain boundary engineering of the intergranular composition are difficult to fabricate and might not survive in high temperature environments. In contrast, a bulk polycrystalline composite of three chemically and structurally distinct phases will generate heterointerfaces by using simple sintering techniques (Fig. 4). These heterointerfaces are shown to enhance the radiation damage tolerance of the most susceptible phase. In this case, submicron Al_2_O_3_ (not even nanocrystalline!) demonstrates high damage tolerance just by being incorporated in a composite system. This approach to developing more radiation tolerant materials is applicable for a wide range of compositions.

## Methods

### Sample preparation

Single crystals of Al_2_O_3_ with the densest planes oriented to the surface (0001) was purchased from MTI [CA, USA]. Bulk samples of single phase and three-phase compositions of YSZ (8 mol% Y_2_O_3_) [Tosoh, Tokyo, Japan], α-Al_2_O_3_ [Taimei, Tokyo, Japan supplied by Pred materials, USA] and MgAl_2_O_4_ [Baikalox, TX] were prepared by spark plasma sintering (SPS)^27^. Before SPS, the powders were milled in a high speed planetary ball mill to reduce the prevalence of agglomerates and to mix well. Single phase samples were sintered by SPS at 950 °C for 5 minutes using a ramp rate of 10 °C/min under 100 MPa and three-phase samples were sintered at 950 °C for 5 minutes using a ramp rate of 100 °C/min under 100 MPa. For irradiation, 13 mm diameter SPS samples were cut into pellets with ~1 mm thickness. Pellets were annealed at 1000 °C for 1 hour in air after SPS to remove carbon contamination, a by-product of the graphite foil and dies used in the SPS process and then polished on one side to 60 nm finish. After annealing, SPS samples had grain sizes of 200–290 nm and relative densities greater than 90%. Certain SPS three-phase samples were further annealed at 1350 °C for 20 hours to grow the average grain size to ~690 nm.

### Irradiation condition

The polished side of the samples was irradiated with 4 MeV Si^2+^ beam at 650 °C under a vacuum of 5 × 10^–5^ Pa at the Ion Beam Materials Laboratory (UT-ORNL IBML) at University of Tennessee, Knoxville^28^. The temperature was chosen to better simulate the conditions in a nuclear reactor and to promote the diffusion of point defects to form dislocation loops that could be readily identified. The samples were tilted 7 degrees with respect to the incident ion beam to avoid ion channeling. Adjustable beam slits were used to define an irradiation area covering the entire sample surface. The ion beam was defocused and wobbled in the horizontal and vertical directions over a wider area with the aim of defining a homogeneous irradiated region. Beam homogeneity was within 10% throughout the irradiated area, which was validated by checking the ion beam induced luminescence (IL) on Al_2_O_3_ (used as a scintillator) monitored with a CCD camera. Irradiations were performed with 4 MeV Si^2+^ using low beam current densities in the range of 3.3 nA/mm^2^ to avoid beam heating and charge accumulation on the samples. The ion flux of 1 × 10^12^ cm^−2^/s and fluence of 1 × 10^16^ ions/cm^2^ were kept constant throughout each irradiation. Displacement per atom, ion range and energy loss as a function of depth into the sample were calculated using the Stopping and Range of Ions in Matter (SRIM) binary collision approximation (BCA) software in Kinchin-Pease mode^29,30^. The assumed displacement energies are: 20 eV for aluminum and 50 eV for oxygen in Al_2_O_3_^31^, 40 eV for magnesium, 40 eV for aluminum and 40 eV for oxygen in MgAl_2_O_4_^18^ and 80 eV for zirconium and yttrium and 120 eV for oxygen in YSZ^32^. The calculated stopping powers (electronic and nuclear), ion range and atomic displacements including all the host ions (dpa) as a function of depth into the sample are shown in Fig. 1. The steady state temperature of the sample surface was measured via a K-type (chromel-alumel) thermocouple (TC). The thermal couple was attached to the sample surface using a clip. More details on the experimental apparatus are provided elsewhere^28^.

### Characterizations

Samples were prepared for TEM imaging using Ga focused ion beam (FIB) on Quanta dual beam SEM/FIB (FEI, Hillsboro, OR). Lamellas were lifted out with OmniProbe 200 (Oxford Instruments, Abingdon, United Kingdom) and attached on Omniprobe Lift-out grids (TED PELLA, INC., CA, USA) on the V position to minimize bending of the foil. An ion beam accelerating voltage of 5 keV with a beam current 16 pA was used for the final polish. The average thick ness of each TEM sample is ~50 nm, confirmed by SEM. TEM samples were imaged with a transmission electron microscope (TEM) [Philips CM-20, Hillsboro, OR] with 200 keV using a LaB_6_ beam source. Some of the images are taken at a high magnification and patched together to make a montage to retain high enough resolution to resolve dislocation loops. Multiple TEM samples were prepared and observed with different tilts for the composite materials. Dislocation loop areal concentration at each depth was calculated from TEM images (~5 × 5 μm^2^) divided into 200 × 1800 nm^2^ sections with random tilts. Standard deviation was generated from the accumulated concertation from each section.

## Electronic supplementary material

Supplementary information

## Data Availability

The datasets generated during and analyzed during the current study are available from the corresponding author on reasonable request.
