# Supplementary material for: Improved high temperature radiation damage tolerance in a three-phase ceramic with heterointerfaces
Source: Sci Rep. 2018 Sep 18;8:13993. doi: 10.1038/s41598-018-31721-x (PMC6143604; doi:10.1038/s41598-018-31721-x)
Supplement: Supplementary file 1 — Supplementary information [file 41598_2018_31721_MOESM1_ESM.docx]

**Improved high temperature radiation damage tolerance in a three-phase ceramic with heterointerfaces**

Kenta K. Ohtaki^1^, Maulik K. Patel^2,3^, Miguel L. Crespillo^3,4^, Keyur K. Karandikar^5^, Yanwen Zhang^3,4^, Olivia A. Graeve^5^, and Martha L. Mecartney^1*^

^1^Department of Chemical Engineering & Materials Science, University of California, Irvine, Irvine, CA 92697-2575, USA.

^2^Department of Mechanical, Materials & Aerospace Engineering, University of Liverpool, Liverpool, L69 3BX, UK.

^3^Department of Materials Science and Engineering, University of Tennessee, Knoxville, Knoxville, TN 37996-2100, TN USA.

^4^Ion Beam Materials Laboratory, University of Tennessee, Knoxville, Knoxville, 37996, TN USA

^5^Department of Mechanical and Aerospace Engineering, University of California, San Diego, San Diego, CA 92093-0411, USA.

^*^Correspondence to: Martha L. Mecartney; [martham@uci.edu](mailto:martham@uci.edu)

Supplementary Information

Calculation of residual stress at grain boundaries and interfaces

In a simple two-layer composite, of different materials A and B with elastic modulus and thermal expansion coefficient of *E*_A_, *α*_A_ and *E*_B_, *α*_B_, the thermal expansion of the composite *α*_C_ is

$\alpha_{C}=(\alpha_{A}+\alpha_{B})/2$­ (1)

using the rule of mixture. Assuming that the two layers were bound at high temperature and cooled down (*ΔT*). If the two materials were NOT bonded, the shrinkage for the material A,*ε*_A_, would be

$\varepsilon_{A}=\Delta T\alpha_{A}$ (2)

And for the material B:

$\varepsilon_{B}=\Delta T\alpha_{B}$ (3)

While the shrinkage of the composite is

$\varepsilon_{C}=\Delta T\alpha_{C}$ (4)

When *ε*_A_>*ε*_B_, *ε*_A_>*ε*_C_>*ε*_B_ and A experiences compressive stress as it cannot contract as much

$\sigma_{A}=E_{A}(\varepsilon_{A}-\varepsilon_{C})$ (5)

And material B experiences tensile stress

$\sigma_{B}=E_{B}(\varepsilon_{B}-\varepsilon_{C})$ (6)

From Equations (4) and (1),

$\varepsilon_{C}=\Delta T(\alpha_{A}+\alpha_{B})/2$ (7)

Thus from (5)-(7) we can generate the residual stress in each phase, given by

$$\begin{matrix} \sigma_{A}=E_{A}(\varepsilon_{A}-\varepsilon_{C})=E_{A}(\Delta T\alpha_{A}-\Delta T(\alpha_{A}+\alpha_{B})/2)=E_{A}\Delta T(\alpha_{A}-\alpha_{B})/2 \\ \sigma_{B}=E_{B}\Delta T(\alpha_{B}-\alpha_{A})/2 \end{matrix}$$

Hence the larger the difference in thermal expansion coefficients (Table S.1.), the greater the residual stress that will develop at a heterointerface. The residual compression stress reaches maximum at a grain boundary between a YSZ/a-axis Al_2_O_3_ and MgAl_2_O_4_/a-axis Al_2_O_3_; 280-303 MPa (Table S.2.). The calculated maximum residual stress in hetero interfaces associated with Al_2_O_3_ grains in a three-phase sample is over 3 times larger than the maximum residual stress between Al_2_O_3_ grains with coincident a-axis and c-axis; 84 MPa. Obviously, the exact residual stress depends on both the composition and the orientation of each grains, but this calculation shows that overall there is a higher probability of higher stresses at YSZ/a-axis Al_2_O_3_ and MgAl_2_O_4_/a-axis Al_2_O_3_heterointerfaces than at Al_2_O_3_/Al_2_O_3_ grain boundaries.

**Table S. 1** Thermal expansion and elastic modulus of each phase

| **Material** | **Thermal expansion (× 10^-6^/°C)** |
| --- | --- |
| 8YSZ | 11.1 at 650°C ^1^ |
| Al_2_O_3_ *a*-axis | 7.1 at 650°C ^2^ |
| Al_2_O_3_ *c*-axis | 8.3 at 650°C ^2 ­^_­_ |
| MgAl_2_O_4_ | 11.4 at 648°C ^3^ |

**Table S. 2** Stress induced by the differences in thermal coefficients in each grain with different interfaces when cooled down from 950°C to 650°C. Negative stress indicates compressive stress.

| **Interfaces** | **Grain Orientation** | **Maximum Induced Stress**  **(MPa)** |
| --- | --- | --- |
| Al_2_O_3_ a-axis\|Al_2_O_3_ c-axis | Al_2_O_3_ a-axis | -84 |
|  | Al_2_O_3_ c-axis | 97 |
| Al_2_O_3_ a-axis\|YSZ | Al_2_O_3_ a-axis | -281 |
|  | YSZ | 140 |
| Al_2_O_3_ a-axis\|MgAl_2_O_4_ | Al_2_O_3_ a-axis | -303 |
|  | MgAl_2_O_4_ | 204 |

**References**

1. Zhang, Y. *et al.* Influence of Gd_2_O_3_ and Yb_2_O_3_ Co-doping on Phase Stability, Thermo-physical Properties and Sintering of 8YSZ. *Chin. J. Aeronaut.* **25,** 948–953 (2012).

2. Munro, M. Evaluated Material Properties for a Sintered alpha-Alumina. *J. Am. Ceram. Soc.* **80,** 1919–1928 (1997).

3. Singh, H. P., Simmons, G. & McFarlin, P. F. Thermal expansion of natural spinel, ferroan gahnite, magnesiochromite and synthetic spinel. *Acta Crystallogr. Sect. A* **31,** 820–822 (1975).
